# High Risks of HIV Transmission for Men Who Have Sex with Men — A Comparison of Risk Factors of HIV Infection among MSM Associated with Recruitment Channels in 15 Cities of China

**DOI:** 10.1371/journal.pone.0121267

**Published:** 2015-04-02

**Authors:** Jinlei Qi, Dapeng Zhang, Xiaojing Fu, Chengmei Li, Sining Meng, Min Dai, Hui Liu, Jiangping Sun

**Affiliations:** 1 National Center for AIDS/STD Control and Prevention, Chinese Center for Disease Control and Prevention, Beijing, China; 2 Bill & Melinda Gates foundation China Office, Beijing, China; Fudan University, CHINA

## Abstract

**Objective:**

While the HIV epidemic varies greatly by region and population group throughout China, the HIV incidence among men who have sex with men (MSM) continues to rise at an alarmingly fast pace. We seek to analyze the risk factors associated with HIV infection among MSM recruited from different channels in large urban centers across China, in an attempt to shed light on the design of future targeted intervention strategies.

**Methods:**

A total of 33,684 MSM from 14 cities and one province were recruited from July to December 2011. Demographic (e.g. age, marital status, education) and behavioral (e.g. condom use, HIV testing history) data were collected using information collection cards. Blood samples were also collected to test for HIV and Syphilis.

**Results:**

Participants were recruited from five different channels, and all demonstrated distinct characteristics. The overall rate of positive HIV screening was 6.27% and the rate of syphilis infection was 6.50%. Participants recruited from bathhouses had the highest HIV (11.80%) and syphilis infection rates (11.20%). Participants who were infected with syphilis had the highest HIV-positive screening rate (13.75%; 95% CI OR, 2.33-3.06). living in the southwest region of the country (11.64%; OR=2.76, 95%CI OR 2.19-3.47), Being >20 years of age (P<0.001), living in the southwest region of the country (OR=2.76, 95%CI 2.19-3.47), not having sex with female over the previous 3 months (OR=1.27, 95%CI 1.09-1.48), no condom use during the last anal intercourse (OR=1.54, 95%CI 1.39-1.70) and other factors were all associated with a higher probability of having an HIV-positive test result.

**Conclusion:**

Depending on the way they are recruited, more targeted interventions are required to prevent the spread of HIV/AIDS among MSM with different characteristics and behaviors. Results from this study could provide evidence for researchers to conduct further studies and policy-makers to establish more effective and strategic interventions for MSM in China.

## Introduction

Epidemiological data in many countries have shown that the HIV epidemic among men who have sex with men (MSM) has become one of the most important priorities in the fight against HIV/AIDS [1–4]. In the United States, MSM account for 57% of new infections in 2008 [5]. High HIV infection rates among MSM have also been reported in Africa, part of Southeast Asia. Beyrer *et al* reported that HIV incidence among MSM in 15 countries including Australia, Canada, Brazil, Argentina, Panama, China, and Thailand, continue to show sustained epidemic patterns, and no downward trend [1].

China is also facing the same challenge in HIV transmission among MSM. Available data indicate that the HIV epidemic among MSM is rapidly expanding [6]. The proportion of homosexual transmission among reported HIV cases increased from 14.7% in 2009 to 17.4% in 2011. For example, HIV incidence in Yunnan Province was 3.5 (95% CI 1.8–6.2) cases /100 person-years [7]. Chongqing City reported that its HIV incidence were 8.1, 9.1, and 9.4 cases /100 person year for 2006, 2007, and 2008, respectively [8].

Reports show that MSM account for only 2–4% of the Chinese adult male population[9]. However, since China is the world's most populous country, the absolute number of the MSM population is large. At present, MSM in China seek their sexual partners via public venues (e.g. parks and public toilets), entertainment establishments (e.g. gay bars, bathhouses, and saunas), and gay websites and internet chat tools (e.g. QQ, MSN, online chat rooms). These venues and channels in which MSM seek homosexual partners have also been the major platforms where health education, behavioral interventions, and HIV testing and counseling promotion take place.

Due to the existing stigma against MSM in China, this population remains relatively hidden. One of the key prevention strategies targeting MSM in China is to promote HIV testing and counseling to increase case finding [10, 11]. Since community-based organizations (CBOs) have established relatively more trusting, long-term relationships with the MSM population, they play a significant role in the promotion and implementation of HIV interventions [12].

Previous studies have presented conflicting findings on MSM’s sexual behavior pattern, especially those recruited from gay bathhouses. Some studies indicated that gay bathhouses have served as sites for HIV prevention from the early days of the AIDS epidemic[13]. MSM who often visit the bathhouses are more likely to engage in high-risk sexual behaviors than those who seek sexual partners from other venues[14]. Results from the Urban Men’s Health Study confirmed that men who engage in risky sexual behaviors are significantly more likely to go to bathhouses than men who do not engage in high-risk behaviors. However, other studies suggest that high-risk sexual behaviors are not that common within the bathhouse setting[15, 16]. It was found that MSM who find homosexual partners online are more likely to engage in unprotected anal intercourse[17].

Thus, there is a need to conduct multiple-venue research to analyze the risks of acquiring HIV infection among MSM recruited from different channels. This study aims to analyze characteristics and behaviors of MSM who were screened HIV-positive from the different recruitment channels. Results from the study could potentially inform the development of future venue-based interventions to help reduce the spread of HIV among MSM.

## Methods

### Study Procedures

Data were collected from 15 project sites included 14 cities and one province: Beijing, Shanghai, Tianjin, Chongqing, Harbin, Shenyang, Qingdao, Xi’an, Nanjing, Wuhan, Hangzhou, Changsha, Kunming, Guangzhou, and Hainan Province.

Two recruitment strategies were used to mobilize the MSM community to join the study. First, the CBOs offered a behavioral intervention to the local MSM community and encouraged them to test at CBO offices or fixed venues, or visiting the local CDC for HIV screening and syphilis testing. Then, participants who were screened HIV-positive were at the local CDC or referred to designated hospital for confirmatory testing, CD4 testing, and other services. Participants were asked to fill out a short survey on the local CDC and CBO website before receiving the testing service, documenting information on socio-demographics, sexual behavior, condom use, and HTC history.

### Ethical Approval

The goal of the project was to improve HIV/AIDS case detection among MSM. The Institutional Review Board (IRB) of the National Center for AIDS/STD Control and Prevention (NCAIDS) approved all informed consent forms that were used in the project. 97% of the subjects (32673) provided their signed informed consent and 3% (1011) provided verbal consent, and there was no different efficacy between written and verbal informed consent(In the process of providing verbal consent, the investigators explained the purpose, the content, risks and benefits of this study, and the rights in participating in the research. If applicable, subjects also signed their names on the first page of the questionnaire). All subjects were given a unique anonymous identification number for privacy protection. All of the above procedures, including the verbal informed consent, were reviewed and approved by the NCAIDS IRB.

### Recruitment Methods

We adopted a diverse range of CBO-based recruitment methods for our study. Some CBOs focused on recruiting MSM for intervention and testing mobilization activities on the internet and via social media tools. Some CBOs conducted interventions in more traditional settings such as gay bathhouses, saunas, gay bars and clubs, parks, and public toilets. They offered interventions and encouraged testing through peer education and outreach services. Many MSM sought testing services at CBO offices through word-of-mouth. We used a combination of these different approaches mentioned above to recruit participants in the 15 project sites.

### Data Collection

Each MSM who was tested for HIV was asked to fill out a questionnaire. The questionnaire included two parts. Part One collected information on social demography, sexual behaviors, condom use, and HIV testing history. It was completed either by the participant himself or by the CBOs’ staff while he/she interviewed the participant. Part Two consisted of HIV/Syphilis screening and testing information, this was collected by the local CDCs. Information collected from the survey was then entered in a web-based data collection tool. Each questionnaire was linked with a unique identification number.

### HIV Screening Services

Two models of HIV testing promotion among MSM were implemented in the 15 project cities and province.

#### Model 1: CBO + Rapid Test

The ‘CBO + Rapid test’ model consisted of the following three testing scenarios: (1) CBOs directly provided HIV rapid tests to MSM with technical support from the local CDCs at the CBO office or fixed permanent location; (2) CBOs provided on-site rapid tests at traditional venues where MSM gather such as gay bathhouses or gay bars, also with technical support from the CDCs; (3) MSM were referred to the local CDCs for HIV rapid testing after the CBOs’ outreach intervention [18].

#### Model II: CBO + ELISA

In this model, MSM were referred to local CDCs and screened for HIV using an ELISA test after CBOs’ outreach intervention[18]. A Determine Finger-prick Rapid Test (Abbott Laboratories, USA) and an ELISA screening test (brand varies by site) were used together as part of the screening package.

### Syphilis Testing Services

**Fig 1:**
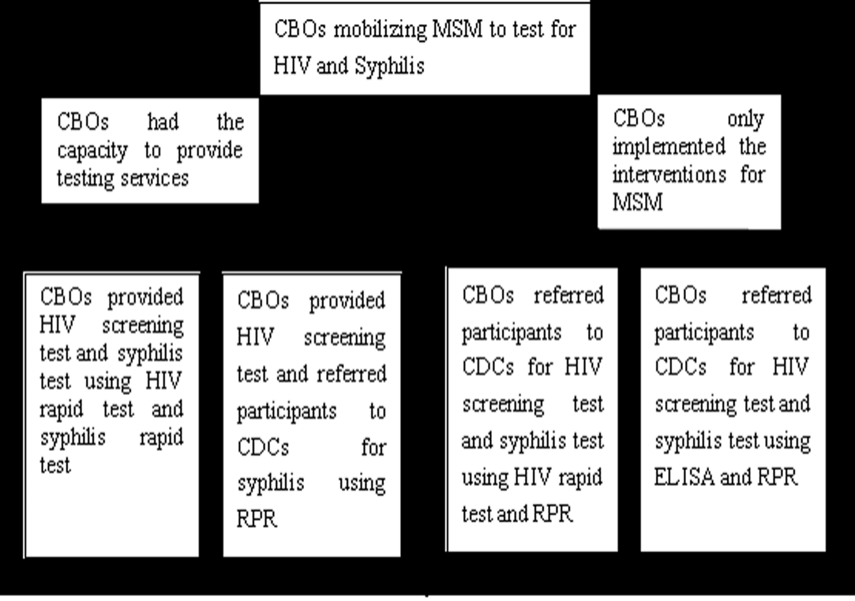
HIV screening and syphilis testing procedures.

Syphilis testing was offered to each MSM who agreed to be tested. The testing process was similar to HIV testing (Fig 1).

#### Model 1: CBO + Rapid Test

(1) CBOs directly provided syphilis rapid tests for MSM at the CBO office or at a fixed permanent location. (2) CBOs provided on-site rapid tests at MSM gathering areas such as gay bathhouses or gay bars. Local CDC staff provided technical support to the CBOs with rapid testing.

#### Model II: CBO + RPR

(1) Blood samples were collected from MSM participants by the CDC staff at the CBO Office or MSM gathering areas. Blood samples were then delivered to the local CDC for testing. (2) MSM were referred to the local CDC or designated hospital for syphilis testing. Blood samples were tested using the Rapid Plasma Reagin (RPR) test kit (Kehua Biostix Inc, Shanghai, China). If the screening result was positive, then subjects would receive a passive particle agglutination test for detection of Antibodies to Treponemapallidum (TPPA, FUJIREBIO INC, Tokyo). Syphilis infection was confirmed if RPR/Rapid Test and TPPA tests were both positive.

### The Classification of Five Groups in This Study

Based on the CBO recruitment channels, participants were divided into five groups (bathhouse, gay bar, internet, park/ public toilet, and other.). The group recruited from the internet included subjects who received interventions and testing mobilization through web-based tools such as gay websites, QQ groups, or mobile instant message tools. The fifth group recruited via “other channels” included those recruited from various CBO campaigns or from the National HIV/AIDS comprehensive intervention program.

### Data Analysis

Chi-square tests were used to assess socio-demographic and behavioral differences between each group. Results of HIV and syphilis tests were also compared. Logistic regression models were used to test the robustness of these differences. The models were also used to calculate both unadjusted and adjusted odds ratios (ORs) and Confidence Intervals(CIs) for predictor variables. Multiple logistic regression was used to adjust possible confounders such as sexual behaviors, condom use history, HTC history, and screening type. The datasets from the bathhouse and internet group (both have high risks for HIV infection) were analyzed separately using multiple logistic regression. Missing values were regarded as separate categories for clarity, but otherwise were not treated differently. Complete cases were used when running each regression model. CIs of HIV-positive screening rate were calculated by approximating the binomial distribution with a normal distribution. All P-values were 2-sided. Data analysis was performed using SAS version 9.2 for Windows x64.

## Results

### Study Population Characteristics

A total of 34,849 MSM participated in the survey from July to December 2011. Participants were excluded from data analysis (1,165, 3.34%) if they did not receive HIV tests, or engage in anal intercourse during the six months prior to the survey. Participants were also excluded if they completed less than 80.00% of the key questions in the survey.

Among 33,684 MSM that were included in the analysis, 2,518 (7.48%) were recruited from gay bathhouses, 4,829 (14.34%) from gay bars, 6,316 (18.75%) via the internet, 2,061 (6.12%) from parks/ public toilets, and 17,960 (53.31%) from other venues. As shown in Table 1, the age of subjects were 30.96±9.57, most participants were between 21 and 30 of age (15324, 45.49%), unmarried (22,648, 67.24%), had an education level of high school and above (26,688, 79.23%), and were local residents (28,918, 85.85%).

**Table 1:** Demographic Characteristics and Sexual Behavior for MSM by Different Recruitment Channels in 15 project cities, Jul to Dec, 2011.

Variables	Bathhouse	Gay bars	Internet	Parks/Toilet	Others	Total
	n(%)	n(%)	n(%)	n(%)	n(%)	n(%)
Age^a^						
≤20	70(2.78)	195(4.04)	279(4.42)	58(2.81)	995(5.54)	1597(4.74)
21–30	756(30.02)	2075(42.97)	3373(53.40)	693(33.62)	8427(46.92)	15324(45.49)
31–40	620(24.62)	1350(27.96)	1232(19.51)	427(20.72)	3534(19.68)	7163(21.27)
41–50	436(17.32)	480(9.94)	312(4.94)	261(12.66)	1357(7.56)	2846(8.45)
≥51	179(7.11)	182(3.77)	84(1.33)	305(14.80)	526(2.93)	1276(3.79)
Unknown	457(18.15)	547(11.33)	1036(16.40)	317(15.38)	3121(17.38)	5478(16.26)
Marital Status^a^						
Single	1237(49.13)	3127(64.75)	4850(76.79)	999(48.47)	12435(69.24)	22648(67.24)
Married	920(36.54)	1249(25.86)	644(10.20)	586(28.43)	3099(17.26)	6498(19.29)
Divorced or widowed	108(4.29)	203(4.20)	112(1.77)	155(7.52)	703(3.91)	1281(3.80)
Living with male partner	147(5.84)	162(3.35)	356(5.64)	111(5.39)	1531(8.52)	2307(6.85)
Unknown	106(4.21)	88(1.82)	354(5.60)	210(10.19)	192(1.07)	950(2.82)
Education Levels^a^						
Secondary school or less	636(25.26)	797(16.50)	491(7.77)	890(43.18)	3639(20.26)	6453(19.16)
Completed High school or vocational school	966(38.36)	2301(47.65)	1653(26.17)	699(33.92)	4908(27.33)	10527(31.25)
College or higher	879(34.91)	1683(34.85)	3799(60.15)	460(22.32)	9340(52.00)	16161(47.98)
Unknown	37(1.47)	48(0.99)	373(5.91)	12(0.58)	73(0.41)	543(1.61)
Current Address^a^ ^b^						
In project areas	1776(70.53)	3790(78.48)	5846(92.56)	1580(76.66)	15926(88.67)	28918(85.85)
Outside project areas	705(28.00)	995(20.60)	438(6.93)	471(22.85)	1881(10.47)	4490(13.33)
Unknown	37(1.47)	44(0.91)	32(0.51)	10(0.49)	153(0.85)	276(0.82)
Region^a^ ^c^						
Northeast Region	475(18.86)	287(5.94)	173(2.74)	342(16.59)	694(3.86)	1971(5.85)
North Region	385(15.29)	1999(41.40)	2624(41.55)	673(32.65)	5748(32.00)	11429(33.93)
Northwest Region	53(2.10)	4(0.08)	716(11.34)	19(0.92)	2452(13.65)	3244(9.63)
East Region	1049(41.66)	1348(27.91)	1299(20.57)	284(13.78)	4059(22.60)	8039(23.87)
South Central Region	473(18.78)	420(0.87)	503(7.96)	614(29.79)	3116(17.35)	5126(15.22)
Southwest Region	83(3.30)	771(15.97)	1001(15.85)	129(6.26)	1891(10.53)	3875(11.50)
Number of Male Sexual Partners^a^						
0	180(7.15)	475(9.84)	214(3.39)	48(2.33)	1053(5.86)	1970(5.85)
1	824(32.72)	1325(27.44)	2630(41.64)	789(38.28)	6980(38.86)	12548(37.25)
≥2	1492(59.25)	2598(53.80)	3356(53.13)	1040(50.46)	9883(55.03)	18369(54.53)
Unknown	22(0.87)	431(8.93)	116(1.84)	184(8.93)	44(0.24)	797(2.37)
Sex with Female in Past 3 Months^a^						
Yes	498(19.78)	812(16.82)	433(6.86)	477(23.14)	2326(12.95)	4546(13.50)
No	1107(43.96)	2581(53.45)	3402(53.86)	976(47.36)	13882(77.29)	21948(65.16)
Unknown	913(36.26)	1436(29.74)	2481(39.28)	608(29.50)	1752(9.76)	7190(21.35)
Used Condom in Past 3 Months^a^						
Yes	1048(41.62)	2780(57.57)	4003(63.38)	1204(58.42)	7412(41.27)	16447(48.83)
No	1188(47.18)	1537(31.83)	2013(31.87)	795(38.57)	9385(52.26)	14918(44.29)
Unknown	282(11.20)	512(10.60)	300(4.75)	62(3.01)	1163(6.48)	2319(6.88)
Used Condom at Last Anal Sex^a^						
Yes	1463(58.10)	3215(66.58)	4713(74.62)	1421(68.95)	10616(59.11)	21428(63.61)
No	759(30.14)	1116(23.11)	1332(21.09)	588(28.53)	6182(34.42)	9977(29.62)
Unknown	296(11.76)	498(10.31)	271(4.29)	52(2.52)	1162(6.47)	2279(6.77)
Ever Tested for HIV Before^a^						
Yes	1178(46.78)	3137(64.96)	3315(52.49)	744(36.10)	7299(40.64)	15673(46.53)
No	1340(53.22)	1692(35.04)	3001(47.51)	1317(63.90)	10661(59.36)	18011(53.47)
Screening Test Type^a^						
ELISA	632(25.10)	3024(62.62)	2944(46.61)	1129(54.78)	9203(51.24)	16932(50.27)
Rapid Test	1886(74.90)	1805(37.38)	3372(53.39)	932(45.22)	8757(48.76)	16752(49.73)
Overall	2518	4829	6316	2061	17960	33684

^a^ **Men from 5 recruited channels were significant difference in all of the above indicators.**

^b^ Current Address: ‘In project areas’ refers to MSM who always living in the projected cities. ‘Outside project areas’ refers to MSM who always living in other cities (not projected cities).

^c^Region[3]: Northeast Region(Harbin and Shenyang), North Region(Beijing and Tianjin),Northwest Region(Xi’an),East Region(Shanghai,Qingdao, Nanjing, and Hangzhou),South Central Region(Wuhan, Changsha, Guangzhou, and Hainan Province), Southwest Region(Chongqing and Kunming).

### Different Characteristics between Groups

Table 1 presents the distribution of demographic characteristics, sexual behaviors, and screening test types by recruitment methods. All results were significantly different. Of the men recruited from gay bathhouses, the age were 35.33±10.62, 17.32% were 41 to 50 years of age, 36.54% were married, and 28.00% were visitors; 59.25% had two or more homosexual partners, 41.62% used condoms during the previous three months, and 58.10% used a condom during their last sexual activity. Of the MSM who received an HIV test in the bathhouse group, 74.90% were tested using a rapid test.

The age of MSM recruited via internet was 28.84±7.34. The majority of them were 20 to 30 years old (53.40%), single (76.79%), had a college or higher educational degree (60.15%), and were local residents (92.56%).

In the parks/public toilet group, the mean age was 36.97±14.02, with 14.80% being over 50 years old. 43.18% reached up to a junior high educational level. 22.85% were visitors, and 63.90% never received HIV testing before the intervention.

### Results of Syphilis/ HIV Screening and Risk Factor Analysis

Table 2 shows the HIV screening and syphilis results by group. MSM recruited from the bathhouses had the highest proportion of HIV screened positive result (11.80%), as well as the highest active syphilis infection rate (11.20%). This was followed by participants recruited via the internet, where the proportion of HIV-positive screening result was 7.90% and the active syphilis infection rate was 6.68%.

**Table 2:** HIV Screening and syphilis test results for Men Who Have Sex with Men by Different Recruitment Channel in 15 project cities, Jul to Dec, 2011.

	****Bathhouse****		****Gay bars****		****Internet****		****Parks/Toilet****		****Others****		****Total****	
	n	%	n	%	n	%	n	%	n	%	n	%
**HIV Screening Test Results** ^**a**^												
** Positive**	297	11.80	241	4.99	499	7.90	136	6.60	939	5.23	2112	6.27
** Negative**	2221	88.20	4588	95.01	5817	92.10	1925	93.40	17021	94.77	31572	93.73
**Syphilis Results** ^**b**^												
** Positive**	282	11.20	216	4.47	422	6.68	166	8.05	1103	6.14	2189	6.50
** Negative**	2002	79.51	4558	94.39	5844	92.53	1831	88.84	16277	90.63	30512	90.58
** Missing/untested**	234	9.29	55	1.14	50	0.79	64	3.11	580	3.23	983	2.92

^a^ Men from 5 recruited channels were significant difference in HIV screening test result χ^2^ = 206.357, p-value<0.001

^b^ Men from 5 recruited channels were significant difference in Syphilis test result χ^2^ = 668.7344, p-value<0.001.

Table 3 shows the distributions of HIV-positive screening test stratified by different factors with unadjusted and adjusted odds ratios (ORs) and CIs for predictor variables. As shown in Table 3, MSM who had syphilis had a higher HIV prevalence (13.75%) than those without syphilis test results and those with syphilis negative results. The gay bathhouse group had the highest HIV prevalence (11.80%). Being >20 years of age (*P<*0.001), living in the southwest region of the country (*OR* = 2.76, 95%*CI* 2.19–3.47), not having sex with female over the previous 3 months (*OR* = 1.27, 95%*CI* 1.09–1.48), no condom use during the last anal intercourse (*OR* = 1.54, 95%*CI* 1.39–1.70), having never tested for HIV prior to the survey(*OR* = 1.13, 95%*CI* 1.02–1.24), receiving a rapid test (*OR* = 1.18, 95%*CI* 1.06–1.31), and having an active syphilis infection (*OR* = 2.67, 95%*CI* 2.33–3.06) were all associated with a higher probability of having an HIV-positive test result. In contrast, having a college degree or higher educational level (*OR* = 0.80, 95%*CI* 0.71–0.91), being a local resident (*OR* = 0.75, 95%*CI* 0.66–0.86), finding sex partners in gay bars (*OR* = 0.41, 95%*CI* 0.34–0.50), or via the internet(*OR* = 0.82, 95%*CI* 0.69–0.97), or at parks/public toilets (*OR* = 0.57, 95%*CI* 0.46–0.72), or via other recruitment methods (*OR* = 0.49, 95%*CI* 0.41–0.57) contributed to lowered chances of HIV infection.

**Table 3:** HIV screening positive rate and Risk Factors for Men Who Have Sex with Men by HIV screening positive in 15 Project cities, Jul to Dec, 2011.

Variables	No.	HIV positive[n(%)]	Unadjusted *OR*(95%*CI*)	*p-value*	Adjusted *OR*(95%*CI*)	*p-value*
Age						
≤20	1597	72(4.51)	1.00		1.00	
21–30	15324	893(5.83)	1.31(1.02,1.68)	0.031	1.35(1.05,1.74)	0.019
31–40	7163	511(7.13)	1.63(1.26,2.10)	<0.001	1.64(1.26,2.14)	<0.001
41–50	2846	237(8.33)	1.92(1.47,2.52)	<0.001	1.72(1.28,2.32)	<0.001
≥51	1276	101(7.92)	1.82(1.33,2.49)	<0.001	1.56(1.11,2.19)	0.011
Unknown	5478	298(5.44)	1.22(0.94,1.59)	0.142	1.00(0.76,1.33)	0.980
Marital sStatus						
Single	22648	1332(5.88)	1.00		1.00	
Married	6498	480(7.39)	1.28(1.15,1.42)	<0.001	1.10(0.95,1.28)	0.201
Divorced or widowed	1281	115(8.98)	1.58(1.29,1.93)	<0.001	1.16(0.93,1.45)	0.198
Living with male partner	2307	138(5.98)	1.02(0.85,1.22)	0.845	0.97(0.81,1.17)	0.775
Unknown	950	47(4.95)	0.83(0.62,1.12)	0.23	0.90(0.64,1.25)	0.516
Education Levels						
Secondary school or less	6453	475(7.36)	1.00		1.00	
Completed High school or vocational school	10527	713(6.77)	0.91(0.81,1.03)	0.145	0.91(0.80,1.04)	0.156
College or higher	16161	909(5.62)	0.75(0.67,0.84)	<0.001	0.75(0.66,0.86)	0.000
Unknown	543	15(2.76)	0.36(0.21,0.60)	<0.001	0.37(0.21,0.66)	0.001
Secondary school or less	6453	475(7.36)	1.00		1.00	
Current Address						
In project areas	28918	1711(5.92)	1.00		1.00	
Outside project areas	4490	377(8.40)	1.46(1.3,1.64)	<0.001	1.42(1.25,1.61)	<0.001
Unknown	276	24(8.70)	1.51(0.99,2.31)	0.054	1.68(1.09,2.59)	0.019
Region^ac^						
Northeast Region	1846	125(6.34)	1.00		1.00	
North Region	10823	606(5.30)	0.83(0.68,1.01)	0.061	1.02(0.82,1.27)	0.856
Northwest Region	3078	166(5.12)	0.8(0.63,1.01)	0.062	1.29(0.99,1.69)	0.061
East Region	7546	493(6.13)	0.96(0.79,1.18)	0.729	1.03(0.82,1.28)	0.823
South Central Region	4855	271(5.29)	0.82(0.66,1.03)	0.083	0.97(0.76,1.23)	0.798
Southwest Region	3424	451(11.64)	1.95(1.58,2.39)	0.000	2.76(2.19,3.47)	0.000
Number of Male Sexual Partners						
0	1970	138(7.01)	1.00		1.00	
1	12548	708(5.64)	0.79(0.66,0.96)	0.017	0.94(0.70,1.25)	0.656
≥2	18369	1208(6.58)	0.93(0.78,1.12)	0.467	1.08(0.81,1.44)	0.614
Unknown	797	58(7.28)	1.04(0.76,1.43)	0.8	1.59(1.05,2.40)	0.029
Sex with Female in Past 3 Months						
Yes	4546	268(5.90)	1.00		1.00	
No	21948	1365(6.22)	1.06(0.92,1.21)	0.409	1.27(1.09,1.48)	0.002
Unknown	7190	479(6.66)	1.14(0.98,1.33)	0.098	1.35(1.13,1.61)	0.001
Used Condom at Last Anal Sex						
Yes	21428	1152(5.38)	1.00		1.00	
No	9977	788(7.90)	1.51(1.37,1.66)	<0.001	1.54(1.39,1.70)	<0.001
Unknown	2279	172(7.55)	1.44(1.22,1.70)	<0.001	1.37(1.05,1.79)	0.022
Ever Tested for HIV Before						
Yes	15673	924(5.90)	1.00		1.00	
No	18011	1188(6.60)	1.13(1.03,1.23)	0.008	1.13(1.02,1.24)	0.015
Screening Test Type						
ELISA	16932	920(5.43)	1.00		1.00	
Rapid Test	16752	1192(7.12)	1.33(1.22,1.46)	<0.001	1.18(1.06,1.31)	0.002
Syphilis Results						
Negative	30512	1738(5.70)	1.00		1.00	
positive	2189	301(13.75)	2.64(2.32,3.01)	<0.001	2.67(2.33,3.06)	<0.001
Missing/untested	983	73(7.43)	1.33(1.04,1.69)	0.022	1.43(1.11,1.85)	0.006
Screening Test Recruitment Channel						
Bathhouse	2518	297(11.80)	1.00		1.00	
Gay bars	4829	241(4.99)	0.39(0.33,0.47)	<0.001	0.41(0.34,0.50)	<0.001
Internet	6316	499(7.90)	0.64(0.55,0.75)	<0.001	0.82(0.69,0.97)	0.022
Parks/Toilet	2061	136(6.60)	0.53(0.43,0.65)	<0.001	0.57(0.46,0.72)	<0.001
Others	17960	939(5.23)	0.41(0.36,0.47)	<0.001	0.49(0.41,0.57)	<0.001
Overall	33684	2112(6.27)	-	-	-	-

As Table 4 showed that MSM recruited from gay bathhouses were found to be associated with a higher probability of having an HIV-positive screening result, we conducted a further regression analysis among gay bathhouse groups. In Table 4, for men recruited from gay bathhouses, being married (*OR* = 1.50, 95% *CI* 1.05–2.13), divorced or widowed (*OR* = 2.31, 95% *CI* 1.33–4.02), and having an active syphilis infection (*OR* = 3.44, 95% *CI* 2.53–4.68) were factors associated with testing HIV-positive. Receiving a rapid test (OR = 1.56, 95% CI 1.10–2.22) was more likely to be associated with a getting positive HIV diagnosis. Having obtained a college degree or higher (*OR* = 0.68, 95% *CI* 0.49–0.96) was a protective factor for men from getting an HIV infection.

**Table 4:** Rate of HIV-positive screening and Risk Factors for Men Who Have Sex with Men for Gay bathhouse groups in 15 Project Cities, Jul to Dec, 2011.

Variables	No.	HIV positive[n(%)]	Unadjusted OR(95%CI)	*p-valu*e	Adjusted OR(95%CI)	*p-value*
Age						
≤20	70	4(5.71)	1.00		1.00	
21–30	756	69(9.13)	1.66(0.59,4.68)	0.341	1.59(0.55,4.59)	0.392
31–40	620	78(12.58)	2.37(0.84,6.7)	0.102	1.74(0.60,5.09)	0.308
41–50	436	65(14.91)	2.89(1.02,8.2)	0.046	1.80(0.60,5.33)	0.292
≥51	179	26(14.53)	2.8(0.94,8.35)	0.064	1.35(0.43,4.25)	0.605
Unknown	457	55(12.04)	2.26(0.79,6.44)	0.128	1.47(0.49,4.39)	0.490
Marital Status						
Single	1237	111(8.97)	1.00		1.00	
Married	920	132(14.35)	1.7(1.3,2.22)	0.000	1.50(1.05,2.13)	0.025
Divorced or widowed	108	24(22.22)	2.9(1.77,4.75)	0.000	2.31(1.33,4.02)	0.003
Living with male partner	147	14(9.52)	1.07(0.6,1.92)	0.826	0.91(0.50,1.67)	0.768
Unknown	106	16(15.09)	1.8(1.02,3.18)	0.041	1.42(0.77,2.63)	0.259
EducationLevels						
Secondary school or less	289	33(11.42)	1.00		1.00	
Completed High school or vocational school	1776	207(11.66)	0.78(0.58,1.04)	0.095	0.78(0.57,1.07)	0.120
College or higher	416	51(12.26)	0.6(0.44,0.82)	0.002	0.68(0.49,0.96)	0.029
Unknown	37	6(16.22)	0.9(0.34,2.37)	0.833	0.94(0.34,2.58)	0.903
Current Address						
In project areas	1776	207(11.66)	1.00		1.00	
Outside project areas	705	84(11.91)	1.03(0.78,1.34)	0.856	0.87(0.65,1.16)	0.328
Unknown	37	6(16.22)	1.47(0.60,3.56)	0.397	1.48(0.59,3.70)	0.403
Number of Male Sexual Partners						
0	180	27(15.00)	1.00		1.00	
1	824	80(9.71)	0.61(0.38,0.97)	0.039	0.77(0.42,1.42)	0.406
≥2	1492	187(12.53)	0.81(0.52,1.26)	0.350	0.88(0.49,1.58)	0.666
Unknown	22	3(13.64)	0.89(0.25,3.23)	0.865	0.94(0.23,3.82)	0.933
Sex with Female in Past 3 Months						
Yes	498	65(13.05)	1.00		1.00	
No	1107	117(10.57)	0.79(0.57,1.09)	0.147	1.03(0.71,1.48)	0.883
Unknown	913	115(12.60)	0.96(0.69,1.33)	0.806	1.19(0.80,1.77)	0.403
Used Condom at Last Anal Sex						
Yes	1463	146(9.98)	1.00		1.00	
No	759	105(13.83)	0.6(0.42,0.86)	0.006	1.32(0.99,1.77)	0.058
Unknown	296	46(15.54)	0.87(0.6,1.27)	0.477	1.25(0.77,2.06)	0.368
Ever Tested for HIV Before						
Yes	1178	134(11.38)	1.00		1.00	
No	1340	163(12.16)	0.93(0.73,1.18)	0.540	1.09(0.84,1.41)	0.521
Screening Test Type						
ELISA	632	50(7.91)	1.00		1.00	
Rapid Test	1886	247(13.10)	1.75(1.28,2.41)	0.001	1.56(1.10,2.22)	0.013
Syphilis Results						
Negative	2002	190(9.49)	1.00		1.00	
Positive	282	79(28.01)	0.77(0.51,1.18)	0.228	3.44(2.53,4.68)	<0.001
Missing/untested	234	28(11.97)	2.86(1.78,4.59)	<0.001	1.38(0.87,2.21)	0.173
Overall	2518	297(11.80)	-	-	-	-

## Discussion

We used a variety of CBO-based recruitment methods to recruit MSM from gay bathhouses, bars, clubs, public toilets, and the internet. Among the 33,684 participants, we found that the overall rate of positive HIV screening was 6.27% (2,112/33,684) and the rate of syphilis infection was 6.50% (2,189/33,6841). Other related factors associated with HIV infections were within our expectation. Our results showed that factors such as old age, living in the southwest region of the country, having engaged in high risk sexual behaviors (i.e. not using condom at last anal sex), having never been tested for HIV prior to the survey, and an active syphilis infection were associated with an increased probability of HIV-positive screening. Having a college degree or above were protective factors. Having excluded the confounding factors, the risks of HIV infection among participants recruited from bathhouses were also higher than other groups. Variability across the five recruitment channels in socio-demography, sexual behaviors, and HIV/syphilis testing information showed that there were more differences than similarities between the five groups.

Our study found that MSM living in the southwest region (Chongqing and Kunming) of the country had a high rate of HIV positive screening, and other researches had shown similar results[3, 14]. It is difficult to explain why the rate was higher than in other regions based on the existing data in our study. Moreover, we noticed that the number of participants recruited by different channels varied by region. We found that the majority of the MSM recruited from gay bathhouses lived in the north, northeast and south central regions of China, while most MSM recruited from gay bars or via the internet came from the northern and eastern regions of the country. MSM recruited from parks/public toilets mostly came from the northeast and the south central region. This could be due to political, economic and cultural difference by region that could potentially have an indirect impact on the MSM population[19]

Previous studies have shown that the HIV prevalence among men who go to gay bathhouses are more than three-fold than that of men recruited from other venues[14]. Subsequently, they would be at a higher risk of HIV and syphilis infection, which is similar to the aforementioned studies.[20]. Men recruited from gay bathhouses tended to be married, older, had lower educational levels, and other factors that would provide opportunities for non-local residents to visit. Bathhouses would as fixed physical venues for visiting MSM to engage in sex. At bathhouses where there is rapid turnover of partners, MSM tend to have more unprotected anal intercourse [21, 22]. Based on the above, further interventions in gay bathhouses need to be strengthened urgently in China.

Also, MSM who were married were more likely to be infected with HIV/AIDS, which implied that their spouses also had greater risk of HIV infection. A study reported that 68% of MSM who knew they were HIV-positive also had sex with their female partners without protection[23]. All of these add to the challenge of preventing HIV transmission among MSM, as well as those who come into close contact with this particular at-risk group.

Bathhouses, which act as venues for sexual encounters with peers, also have the potential to act as locations for HIV testing and counseling promotion. Research indicates that bathhouse-based VCT successfully promote HIV testing among men who have HIV-related risk behaviors and who have been recently or never received HIV testing[24]. In addition, implementing bathhouse-based VCT does not negatively impact the business of the venues[25]. A study suggested that it was difficult to control HIV incidence in MSM networks through behavioral interventions alone (e.g. reductions in extra-primary partnerships). The oral pre-exposure prophylaxis, rectal microbicide and successful AIDS anti-retroviral therapy for HIV-positive MSM all play a crucial role in reducing the chances of HIV infection for men engaging in unprotected receptive anal intercourse[1]. Combined with the aforementioned research, the feasibility of providing oral pre-exposure prophylaxis rectal microbicide in bathhouses or bathhouse-based VCT by using rapid test kits should be studied further.

Our results show that an active syphilis infection is strongly associated with a positive HIV screening result for MSM, which is consistent with findings from previous studies[26]. This means that not only the MSM population should remain the focal point in HIV prevention activities, but also they should be given active treatment for other sexually transmitted infections.

An increasing number of MSM are using the internet (e.g. gay chat-rooms, gay QQ groups) to look for sex partners [17, 27–29]. Previous research reported that 82% had looked for a sex partner on the internet and three-quarters of the 82% had been doing so for more than a year[30]. We did not find, however, that MSM recruited via the internet had a higher risk of HIV infection compared to those recruited via bathhouse. What we did find was that MSM recruited via the internet were younger and had higher education levels. However, the rate of HIV infection for internet-based MSM was found to be higher than that of MSM recruited from gay bars, clubs, and other venues. Some reports indicate that men who seek homosexual partners online are more likely to have unprotected anal intercourse and more likely to become infected with HIV than MSM who do not seek sex online[31]. These evidences indicate that intervention for MSM seeking sexual partners from internet can not be ignored.

It is important to note that the internet is not only a platform for MSM to seek sex partners, but also an information delivery channel. Research shows that most MSM prefer to communicate HIV status and sexual preferences with prospective partners when meeting online rather than in person[32],and they can accept the increasing presence of public health agencies or safe-sex messaging online[33, 34]. They allow health workers into chat rooms and tolerate banners and pop-ups that discuss sexual health. They are open to health discussions (84%) if they meet a health worker in a chat room. This indicates that web-based interventions can be an effective approach to reach more hidden MSM. We advise that policy makers should consider placing a high priority on internet-based public health outreach intervention in the development of future HIV prevention strategies in China.

### Limitations

Several limitations exist in our data. Since the data were extracted from the China-Gates HIV Program, and the program was only conducted in large urban centers, small towns and rural areas were not involved. Thus, the results for these pooled estimates must be interpreted with caution, since the analysis only accounts for sampling variations, and lacks generalizability.

Personal information was collected anonymously and each person was given a unique identification number. Although we only used six months of data (July through December 2011) to minimize the chances of getting repeated questionnaires or HIV screening duplicates, this does not directly exclude survey or testing duplication, which could lead to an underestimation of the actual variances in analyses.

Additionally, administering the questionnaires at the CDC and the CBOs resulted in missing data. Some relevant factors (e.g. drug use, buying and selling commercial sex) were not included in the questionnaire because the program was initially not intended to be a research project.

### Conclusion

Findings from this study could provide evidence for researchers to conduct further studies targeting the MSM population in China. The results can also help policy-makers design and implement more effective and strategic MSM interventions.
